# 

**Published:** 1870-04

**Authors:** 


### Editorial Department.

### Meeting of the American Medical Association.

The American Medical Association will hold its next annual meeting in Washington, May 3d, when it is to be hoped the profession from all parts of the country will be fully represented. In our last issues we published the committees from whom reports may be expected, many of which will be of the greatest interest. In addition to this, considerable medical legislation may be expected, which will be of the greatest importance and should be watched and protected by all interested. It is believed that this meeting will be one of the most interesting and profitable ever held by the association, and we hope that the various societies and institutions of the country will be represented by full delegations.

### Books and Pamphlets Received.

A Practical Treatise on the Diagnosis, Pathology and Treatment of Diseases of the Heart. By Austin Flint, M. D., Professor of the Principles and Practice of Medicine, and of Clinical Medicine, in the Bellevue Hospital Medical College, etc. Second Edition, thoroughly revised and enlarged. Philadelphia; Henry C. Lea.

A Hand-Book of Operative Surgery. By John H. Packard, M. D., one of the Surgeons to the Episcopal Hospital, Secretary of the College of Physicians of Philadelphia, Author of a Manual of Minor Surgery, etc. With fifty-four steel plates and numerous illustrations on wood. Philadelphia; J. B. Lippincott & Co. For sale by Breed & Lent.

The Preventive Obstacle, or Conjugal Onanism. By L. F. E. Bergeret, Physician-in-chief of the Arbois Hospital. Translated from the Third French Edition, by P. De Marmon, M. D. New York; Turner & Mignard, 109 Nassau street.

The Bible in the Public Schools. Opinions of Individuals and the Press, with Judicial Decisions. New York; J. W. Schemerhorn & Co, 14 Bond street. Price twenty-five cents.

Population: Its laws of Increase. By Nathan Allen, M. D., Lowell, Mass.

On the "Sedative" Action of Calomel in Disease. By Frederick B. Lente, M. D., Cold Spring, N. Y.

Valedictory Address to the Graduating Class of Jefferson Medical College, at the Forty-fifth annual Commencement, March 12th, 1870. By J. Aitken Meigs, M. D., Professor of the Institute of Medicine, etc.

Catalogue of the Officers and Students of Lafayette College, for the year 1868—70. Easton, Pa.

Correspondence corcerning a Fatal Case of Placenta Previa. Prepared by Chas. E. Buckingham, M. D., Professor of Midwifery and Medical Jurisprudence in Harvard University.

Reply to Dr. Lewis A. Sayre's Review of Dr. Ruppanner's case of Laryngo-Tracheotomy, to which is added a full account of the great Poisoning case by Partridges at the Fifth Avenue Hotel. By A. Ruppaner, M. D., Physician to the New York Dispensary, for diseases of the Throat and Chest, etc.

Reply to Dr. C. A. Robertson's Review of the Report concerning the last illness of Dr. Alden March. By James McNaughton, M. D., Professor of Theory and Practice in the Albany Medical College.

Proceedings of the Homœpathic Medical Society of the State of Ohio. Fifth Annual Session, 1869.

Intermarriage of Kindred. By Alexander Wilder, M. D., President of the New York State Eclectic Society.

The Technologist: Especially devoted to Engineering, Manufacturing and Building, New York.

The Arts: Devoted to Science and the Arts, Chicago.

The College Courant. Chas. C. Chatfield, A. M., New Haven, Conn.

Catalogue of the Old Colony Nurseries. B. M. Watson, Plymouth, Mass.

Every Saturday. Boston, Fields, Osgood & Co.

